# Challenges in the celiac disease diagnosis; Prague consensus

**Published:** 2017

**Authors:** Gabriel Samasca, Genel Sur, Iulia Lupan, Peter Makovicky, Hugh James Freeman

**Affiliations:** 1 Department of Immunology, , Iuliu Hatieganu University of Medicine and Pharmacy, Cluj-Napoca, Romania; 2 Department of Pediatrics II, Iuliu Hatieganu University of Medicine and Pharmacy, Cluj-Napoca, Romania; 3 Babes-Bolyai University, Department of Molecular Biology and Biotechnology, ClujNapoca, Romania; 4 Czech Centre for Phenogenomics, Institute of Molecular Genetics, Department of Transgenic Models of Disease, ASCR, v.v.i., BIOCEV, Prumyslova 595, 252 42 Vestec, Czech Republic; 5 Department of Gastroenterology, University of British Columbia, Vancouver, British Columbia, Canada

## Introduction

Celiac disease (CD) was initially a real enigma. There were many questions about what immunogenetics markers could influence the immune response (1). Immunogenetic studies provided important contributions to improve the understanding of critical pathogenetic factors at molecular levels (DQ2, DQ8) (2). An important discovery was the recognition of more precise serological markers for CD, specifically anti-endomysial and anti-tissue transglutaminase antibodies leading to an exploration of their increasingly important role in clinical diagnosis of CD (3). Our aim is to explore new and old challenges with unanswered questions in CD diagnosis.

**The last Prague consensus report challenges**

 Re-evaluation of CD diagnostic with intestinal biopsy is required in this consesus (4). However, many patients with CD are still inaccurately diagnosed (or remain undiagnosed), even in referral populations whom were evaluated at tertiary care centers: the diagnosis of CD was confirmed in only 64 patients from 107 patients with previous diagnosis of CD (5). Recently, it was noted that even for some experts, the quality of duodenal bulb biopsies was unsatisfactory. This fact may create confusion and false-positive diagnoses which seem to be increasing in the pediatric population(6). However, histopathological classifications of CD have been debated for decades and some investigators have recently expressed concern regarding specific architectural changes, the degree of change and their relevance: Marsh, Marsh modified (Oberhuber), or Corazza classification. (7, 8). 

 As immunologically-based assays evolve, the authors of this paper believe that serological markers are more useful for screening purposes in CD compared to small intestinal mucosal biopsy alone. At present, the gold standard for diagnosis is still small intestinal mucosal biopsy. Although data is still needed, it is conceivable that serological markers may eventually even become more useful for diagnostic purposes in CD compared to small intestinal mucosal biopsy alone (9). But an obvious serological algorithm is not specified. At present, the IgA anti-tissue transglutaminase antibody test appears to be the most sensitive and specific markers of CD screening (10) but a minority of CD patients are seronegative (11). The main task of gastroenterologists was to recognize untreated CD. In countries with weaker economies (i.e., the so-called developing nations), it is generally believed that the rate of diagnosis is low (12). Controversies remain in areas that include application of screening methods and evaluation of high risk groups for the CD, as well as diagnostic tests that lead to a final diagnosis of the CD (13).

 In addition, a number of immunologically-related challenges in gastroenterology remain for those involved in a CD care. IL-21 was lower in potential CD, than in active CD, so a key role for IL-21 in the progression of mucosal damage in CD needs to be further elucidated (14). Furthermore, HLA DQ8 in Southwest Asia, South America and the Middle East was positive in 49% (n=69) of CD patients and 13% (n=21) of control group, respectively. In conclusion, HLA DQ8 was found to be significantly higher in these geographical regions compared to European countries (15). However, HLA-DQ2 and/or -DQ8 expression: 38.1% in northern, 31.4% in northeastern, and 36.4% in southern India, did not appear to correlate with CD prevalence: 8.53/1,000 and 3.70/1,000 in northern, 4.66/1,000 and 3.92/1,000 in northeastern, and 0.11/1,000 and 1.22/1,000 in the southern India (16). A number of other associated immune-mediated or autoimmune disorders may co-exist with CD, possibly owing to a common genetic background (17). So, novel diagnostic antibodies for CD are neccesary (18). 

## Conclusions

Future research is needed to ease the diagnosis of CD disease. The diagnosis of CD remains a challenge for many gastroenterologists. Critical to this effort in CD, an immune mediated disorder is a close interaction of gastroenterologists and immunologists with a special interest in CD.
